# Characterization of Salinity Tolerance of Transgenic Rice Lines Harboring *HsCBL8* of Wild Barley (*Hordeum spontanum*) Line from Qinghai-Tibet Plateau

**DOI:** 10.3389/fpls.2016.01678

**Published:** 2016-11-10

**Authors:** Wanli Guo, Tianlong Chen, Nazim Hussain, Guoping Zhang, Lixi Jiang

**Affiliations:** ^1^Institute of Crop Science, College of Agriculture and Biotechnology, Zhejiang UniversityHangzhou, China; ^2^Department of Biotechnology, College of Life Science, Zhejiang Sci-Tech UniversityHangzhou, China

**Keywords:** salt tolerance, *HsCBL8*, rice, *Arabidopsis*, *Hordeum spontanum*

## Abstract

Rice is more sensitive to salinity, particularly at its early vegetative and later productive stages. Wild plants growing in harsh environments such as wild barley from Qinghai-Tibet Plateau adapt to the adverse environment with allelic variations at the loci responsible for stressful environment, which could be used for rice genetic improvement. In this study, we overexpressed *HsCBL8* encoding a calcium-sensor calcineurin B-like (CBL) protein in rice. The gene was isolated from XZ166, a wild-barley (*Hordeum spontanum*) line originated from Qinghai-Tibet Plateau. We found that XZ166 responded to high NaCl concentration (200 mM) with more *HsCBL8* transcripts than CM72, a cultivated barley line known for salinity tolerance. XZ166 is significantly different from CM72 with nucleotide sequences at *HsCBL8*. The overexpression of *HsCBL8* in rice resulted in significant improvement of water protection *in vivo* and plasma membrane, more proline accumulation, and a reduction of overall Na^+^ uptake but little change in K^+^ concentration in the plant tissues. Notably, *HsCBL8* did not act on some genes downstream of the rice CBL family genes, suggesting an interesting interaction between *HsCBL8* and unknown factors to be further investigated.

## Introduction

High salinity is one of the most prevalent abiotic stresses that pose severe reduction in plants' growth and productivity of crops such as rice (*Oryza sativa*) grown in coastal and irrigated lands (Martínez-Atienza et al., 2007). Approximately 6% (800 million hectares) of world's total land area has been reported as salt affected (Romeza and Flowers, 2008), mainly contributed by accumulation of salts over time in arid and semiarid regions, salts from oceans brought in by wind and rain, and weathering of the rocks (Rengasamy, 2002). Sodium chloride (NaCl) is usually considered as the major soluble salt in saline soils encountered by plants (Munns and Tester, 2008). The responses of rice to salt stress could involve the regulation of membrane integrity, ionic compartmentation, osmotic adjustment and accumulation of macromolecules (Hu et al., 2012). To cope with deleterious effects of salt stress, efforts have been made to map QTLs that respond to salt stress in rice and barley (Gao and Lin, 2013; Long et al., 2013; Ahmadi-Ochtapeh et al., 2015; Das and Rao, 2015), clone genes based on mapping and transfer elite genes from wild barley into rice (Ellis et al., 2000).

Wild barley (*H. spontanum*), native to Qinghai-Tibet Plateau, has more genetic diversity compared with other wild and cultivated barley species, and the plateau is also considered as the center of the origin for cultivated barley in the oriental region (Dai et al., 2012). Wild barley species thrive best to survive under harsh environments, such as drought (Ahmed et al., 2013) and salt stress (Wu et al., 2013), and could provide elite gene pool for crop improvement (Ellis et al., 2000). Some genes from cultivated barley response to stresses, for example, *HvCBL4* (calcium-sensor calcineurin B-like, CBL), a close homolog of the *AtCBL4*, positively responded to salinity in *Arabidopsis* (Rivandi et al., 2011). Moreover, the overexpression of *HvCBF4* (C-repeat/dehydration-responsive element binding factors, CBF/DREBs) in transgenic rice plants induced tolerance against drought, salinity and low-temperature stresses with normal plant growth (Oh et al., 2007). Maintaining low level of Na^+^ and narrow Na^+^/K^+^ ratio in the cytosol is critical for cellular metabolism and salt tolerance in glycophytes (Qiu et al., 2002). One such example is of tolerant rice subspecies that can exclude Na^+^ from the shoot and maintain a low Na^+^/K^+^ ratio (Mekawy et al., 2015). To date, only *HbCIPK2* from a wild barley species (*H. brevisubulatum*) was reported as a positive regulator of salt and osmotic stress responses, and as a factor to control Na^+^/K^+^ homeostasis in *Arabidopsis* (Li et al., 2012). However, little is known about the functional attributes of the gene(s) in response to abiotic stresses in *H. spontanum* from Qinghai-Tibet Plateau.

Calcium ion (Ca^2+^), the most dynamic second messenger in all eukaryotic organisms, interacts with many binding proteins such as calmodulin (CaM), calmodulin like proteins (CAML), and CBLs. Among these proteins, CBLs, which are small Ca^2+^ binding proteins, mainly interact with CBL-interacting protein kinase (CIPK) as an upstream factor for downstream signaling and in response to several abiotic factors, including salt, cold, drought, proton, reactive oxygen species (ROS), abscisic acid (ABA) and gibberellins (GA) (Thoday-Kennedy et al., 2015). CBLs are most similar to the regulatory B subunit of calcineurin (CNB), a protein phosphatase in animals (Liu and Zhu, 1998). Therefore, the Ca^2+^ binding capacity of the CBL protein is dependent on the number and structure of the elongation factor-hand domain (EF-hand), that could affect the stability of CBL-CIPK complex (Weinl and Kudla, 2009).

The first CBL protein that reacts to salt stress is AtCBL4 (Liu and Zhu, 1998). The active complex of CBL4-CIPK24 complex is activated downstream the Na^+^/H^+^ antiporter (NHX) to induce Na^+^ efflux from roots under high salt conditons (Qiu et al., 2002). The interaction between AtCBL10 and CIPK24 under salt stress also activates the Na^+^ transporter to transfer Na^+^ to the vacuole of shoots or leaves (Quan et al., 2007). *AtCBL1, AtCBL5*, and *AtCBL9* are also involved in the response signaling to salt, drought, osmotic and ROS stress (Thoday-Kennedy et al., 2015). *AtCBL2* and *AtCBL3* could regulate the activity of proton pump V-ATPase on the vacuole membrane to maintain the ion homeostasis in the cell. By contrast, double-*cbl2/cbl3*-mutant plants were found more resistant to high Na^+^ and low K^+^ conditions (Tang et al., 2012). Similar stress responses of CBLs have also been reported in rice *OsCBL8* (Xiang et al., 2007), *Ammopiptanthus mongolicus AmCBL1* (Chen et al., 2011), soybean *GmCBL1* (Li et al., 2012), rape *BnCBL1* (Chen et al., 2012), and tobacco *NsylCBL10* (Dong et al., 2015). Ten CBL proteins have also been discovered in *Arabidopsis*, rice and populous (Albrecht et al., 2001; Kolukisaoglu et al., 2004; Zhang et al., 2008). Eight CBLs have also been detected in maize, sorghum and grape (Weinl and Kudla, 2009). Seven CBLs have been found in canola (Zhang et al., 2014).

*HsCBL8* exhibits a significantly distant phylogenetic relationship between with salt-responsive *AtCBL4*; this relationship indicates that *HsCBL8* can differ from other known plant CBLs (Liu and Zhu, 1998). This finding also shows that *HsCBL8* may function in a mechanism different from that of *AtCBL4*. Some CBL proteins, such as AtCBL1, AtCBL4, AtCBL5, AtCBL8, and AtCBL9 modified through N-myristoylation or S-acylation (Batistic et al., 2012), can acquire functional characteristics in membrane-localized dependent signaling in plant cells. By contrast, CBLs, including AtCBL2, AtCBL3, AtCBL6, and AtCBL7, are characterized by N-myristoylation motifs (MGCXXS/T). In this study, *HsCBL8* was cloned from a wild barely (*H. spontanum*) line XZ166 native to Qinghai-Tibet Plateau and highly tolerant to severe salt stress. This study aimed to investigate the growth performance of transgenic rice plants expressing *HsCBL8* in response to salt stress and to explore the related mechanism.

## Materials and methods

### Plant materials and their growth conditions

The seeds of the Qinghai-Tibetan annual wild barley line XZ166 (*H. spontanum*) and cultivated barley CM72 (*H. vulgare* L. ssp. *Vulgare*), *Arabidopsis thaliana* ecotype Columbia-0 (*Col-0*), and rice Zhonghua11 (ZH11, *Oryza sativa*) were obtained from the Provincial Key Laboratory of Gene Resources, Zhejiang University, Hangzhou, China. The seeds of XZ166 and CM72 were surface sterilized in 5% NaClO for 15 min, rinsed with tap water, and germinated on moistened fine sand in plastic pots (15 cm × 20 cm × 15 cm) for 2 days at 25°C in the dark. The plastic pots with germinated seeds were then transferred to a greenhouse under a 14 h light (420 μmol m^−2^ s^−1^) period at 25°C and 10 h dark period at 23°C daily. We adopted 70% relative humidity and watered the plants with nutrient solution (pH 6.0, mg L^−1^) that contained (NH_4_)_2_SO_4_ (48.2), MgSO_4_ (154.88), K_2_SO_4_ (15.9), KNO_3_ (18.5), KH_2_PO_4_ (24.8), Ca(NO_3_)_2_ (86.17), Fe-citrate (7), MnCl_2_·4H_2_O (0.9), ZnSO_4_·7H_2_O (0.11), CuSO_4_·5H_2_O (0.04), HBO_3_ (2.9), and H_2_MoO_4_ (0.01). The solution was renewed at 6-day intervals, and its value was adjusted to pH 6.0 ± 0.1 using 1 M HCl daily. CaCl_2_ was added with NaCl to maintain a molar ratio of Na^+^:Ca^2+^ = 10:1.

The *Arabidopsis* ecotype *Col-0* and the T_2_ plants (*HsCBL8*_*Promoter*_*-GUS*) were grown for 3 weeks in the greenhouse (16 h light, 110 μmol m^−2^ s^−1^), subsequently transferred to half strength MS liquid media, and allowed to grow for 1 week on the medium. After the plants became adapted to the MS media, different treatments, including 100 μM ABA, 100 mM NaCl, and 20% PEG6000 with 5% sucrose were applied at 4°C for 12 h. Moreover, plants in different developmental stages were used for β-glucuronidase GUS histochemical staining.

The seed dormancy of ZH11 and transgenic plants (*35S-HsCBL8*) was terminated with treatment of 0.5% H_2_O_2_ for 12 h. The seeds were then sterilized in 5% NaClO for 20 min, rinsed with tap water, and germinated on moistened filter papers in an incubator in a 14 h light period (110 μmol m^−2^ s^−1^) and 10 h dark period at 25°C. To calculate the germination rate, 20 rice seeds per biological repeat (four repeats independently) were treated with 125 mM NaCl in 1/4 nutrient solution. Moreover, the solution was renewed daily. Data on germination were collected from the “specific day” until 70 to 80% was reached per treatment for each genotype. Then the germination rate was calculated.

### Molecular cloning of *HsCBL8*

Seedlings of the XZ166 with three leaves were treated with 200 mM NaCl for 48 h, and their roots were selected for RNA extraction. Total RNA was extracted by a kit method (OMEGA, Norcross, GA RNA kit). PrimeScript^TM^ First Strand cDNA Synthesis Kit (Takara, Dalian China) was used to synthesize the cDNA. The genomic DNA of the XZ166 leaves was extracted using the CTAB method. The degenerate primers (Table S1) were designed based on cDNA sequences available on the National Center for Biotechnology Information (NCBI) database with accession IDs AL713904, FJ901265.1, EU085040.1, AB378095.1, XM_002524988.1, DQ907707.1, DQ201200.1, FJ901264.1, and FJ901259.1. The 25 μL PCR reaction mix used for amplification included L-Taq polymerase (Takara, Dalian China) (1.5 units), genomic DNA (25 ng), per primer 10 μM, dNTP (20 mM). PCR amplification was performed as follows: 95°C for 3 min, 10 cycles of 94°C for 30 s, and 60°C for 30 s at −0.5°C per cycle; 72°C for 60 s; and 25 cycles of 94°C for 30 s, 55°C for 30 s, 72°C for 60 s, and 72°C for 5 min. The PCR products were separated by 1.0% agarose gel electrophoresis. We obtained four wild barley CBL sequence candidates; one of which was identified as cultivated barley EST FLbaf27i23 under the Barley Database (http://www.shigen.nig.ac.jp/barley/). The coding sequences (CDSs) of its cDNA and genomic DNA were amplified using primers designed from FLbaf27i23, which contained additional *Xba* I and *BamH* I restriction sites (underlined, Table S1), respectively. The *HsCBL8* promoter was cloned using inverse PCR (IPCR, http://dps.plants.ox.ac.uk/langdalelab/protocols) combined with nested PCR. Genomic DNA (2.5 μg) was cut by *Xho* I and *Cla* I independently; the DNAs ligated by T4 DNA ligase (Takara, Daliang China) were employed for amplification. The primers used are shown in Table S1. The prepared 25 μL PCR reaction mix included LTaq polymerase (Takara, Dalian China) (1.5 units), genomic DNA (50 ng), per primer (10 μM), and dNTPs (20 mM). PCR amplification was performed as follows: 95°C for 3 min, 10 cycles of 94°C for 30 s, 60°C for 30 s, −0.5°C per cycle, and 72°C for 3 min; 25 cycles of 94°C for 30 s, 55°C for 30 s, 72°C for 3 min; and 72°C for 5 min. PCR products were separated by 1.0% agarose gel electrophoresis. Then, the *HsCBL8* promoter region was amplified using primers (Table S1) that contained additional *Hind* III and *Xba* I restriction sites (underlined).

### Analysis of *HsCBL8* CDS and promoter sequences

Nucleotide and protein sequences (Table S2) were analyzed by BioEdit 7.2.5 software (Abbott, Carlsbad, Canada) and NCBI (http://blast.ncbi.nlm.nih.gov/). Amino acid sequence alignment was analyzed by Clustal X, and phylogenetic trees were established by the neighborhood-joining bootstrap method; using the default parameters in the software MEGA 6.06 (http://www.megasoftware.net/index.php). The *cis* elements of the *HsCBL8* promoter sequence (the upstream 1000 bp from the initiation codon) were predicted by PlantCARE online (http://bioinformatics.psb.ugent.be/webtools/plantcare/html/).

### Vector construct and plant transformation

The transgenic *Arabidopsis* plants harboring *HsCBL8*_*Promoter*_*-GUS* were generated according to the methods we previously described (Wang et al., 2014). In brief, a 2160 bp *HsCBL8* promoter and GUS was inserted into *pCAMBIA1300* binary vector. After verification by restrictive digestion and DNA sequencing, the construct was applied to transform wild type plants by floral dipping using *Agrobacteria tumefaciens* strain GV3103. Transgenic plants (T_0_) survived on solid Murashige and Skoog (MS) medium containing 30 mg ml^−1^ hygromycin were further verified by PCR genotyping. T1 and T2 seedlings were selected by PCR genotyping with the primers listed in Table S1.

**Figure 1:**
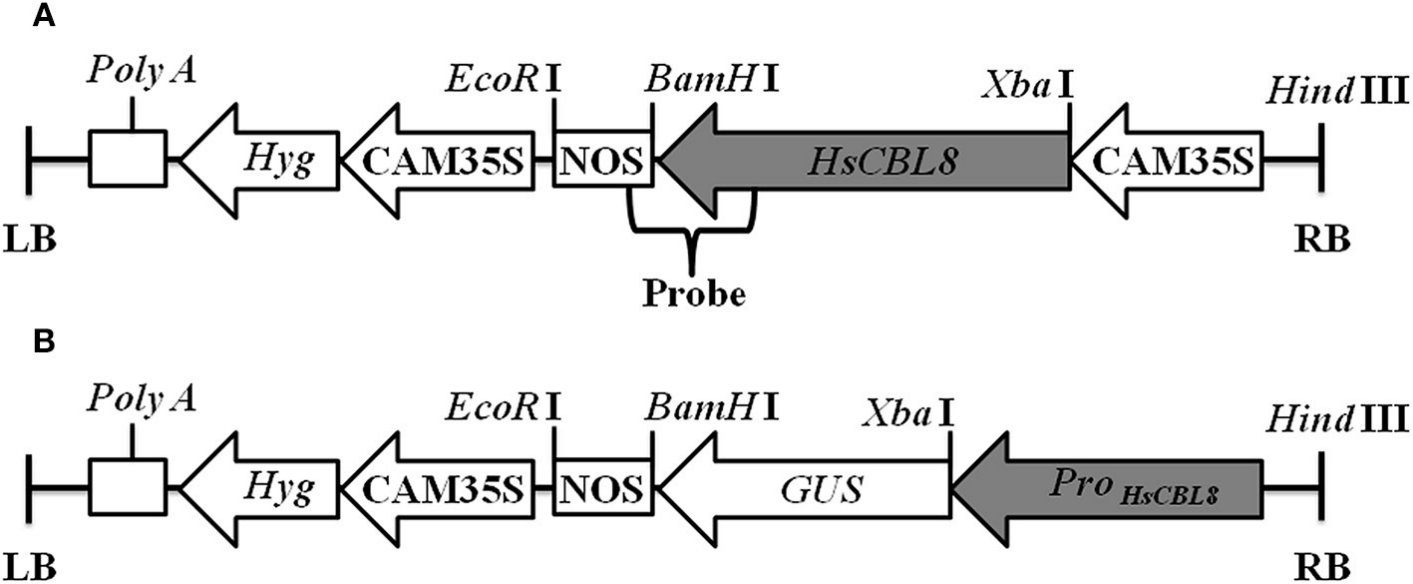
**Linear maps of the molecular constructs ***35S-HsCBL8*** (A)** and ***HsCBL8***_***Promoter***_***-GUS*** **(B)** in the *pCAMBIA1300* vector used for transformation. LB, left border; RB, right border; *Hyg*, Hygromycin B phosphotransferase gene; CAM35S, Cauliflower mosaic virus 35S promoter; NOS, Nos terminator; *GUS*, β-Glucuronidase gene; *ProHsCBL8, HsCBL8* promoter.

*pCAMBIA1300* was modified by inserting the *CaMV 35S promoter-GUS* (*35S-GUS*) region cut from *pBI121* using *Hind* III and *Eco*R I and named as *pCAMBIA1300-35S-GUS*. The genomic DNA of the *HsCBL8* coding region was inserted into the *Xba* I and *BamH* I sites of the *pCAMBIA1300-35S-GUS* plasmid to form expression vector *pCAMBIA1300-35S-HsCBL8* (Figure 1A). This construct was transferred into *Agrobacterium tumefaciens EHA105* and used for rice ZH11 transformation. Callus induction and subculture were performed on the medium supplemented with 2 mg/L 2,4-D, 3% sucrose, and 0.55% agar, named NB medium by Ozawa (2012). After a number of subcultures with an interval of 4 weeks, calli (between 1 and 3 mm in diameter) were used for transformation by co-culture with *EHA105 harboring pCAMBIA1300-35S-HsCBL8*. Then, the co-cultured calli were transferred onto pre-selection medium (NB supplemented with 30 mg/L hygromycin B), and incubated in the dark for 7 days. Subsequently, the differentiated calli were placed on the selection medium (NB with 50 mg/L hygromycin B) for another 14–17 days. The resistant calli were then transferred to pre-regeneration medium (NB supplemented with 2 mg/L 6-BA, 1 mg/L NAA, 5 mg/L ABA and 50 mg/L hygromycin B) and cultured in the dark for 7 days, followed by plant regeneration on medium NB supplemented with 3 mg/L 6-BA, 0.5 mg/L NAA and 50 mg/L hygromycin B, for 14–21 days under a 16 h photoperiod until shoots appeared. Regenerated plants were subsequently transferred into greenhouse and self-pollinated to produce progenies. The transgenic rice was first selected by hygromycin and PCR analysis using primers listed in Table S1, and then confirmed by Southern blot. The 35S region of the *pCAMBIA1300-35S-GUS* plasmid was replaced by the *HsCBL8* promoter (2170 bp before the initiation codon; *HsCBL8*_*promoter*_) to form the vector *pCAMBIA1300-ProHsCBL8-GUS* using *Hind* III and *Xba* I (Figure 1B). This construct was transferred into *A. tumefaciens GV3101* and used for *Arabidopsis* (*Col-0)* transformation by the floral dip method with slight modification. The *Arabidopsis* plants were grown in a greenhouse for about 4 weeks with a cycle of 16 h light and 8 h dark periods before transformation. *GV3101* cells, cultured in Luria–Bertani (LB) medium for 18–20 h at 28°C (optical density at 600 nm [OD_600_]: 1.6–2.0), were collected by centrifugation and re-suspended in infiltration medium (half-strength MS medium, 5% sucrose, 0.044 μM benzylamino purine, and 0.2% Silwet L-77) to an OD_600_ of 0.8–1.0. Plant inflorescences were dipped in the infiltration solution for 2 min, and transferred to a dark chamber in a greenhouse for 2 days, and then cultured under normal conditions. Transgenic plants (T_0_) survived on solid Murashige and Skoog (MS) medium containing 30 mg ml^−1^ hygromycin, were further verified by PCR genotyping. T1 and T2 seedlings were selected by PCR genotyping with the primers listed in Table S1.

### PCR analysis

Both genomic and cDNA were identified. The specific primers (Table S1) from *HsCBL8* genomic DNA were used to separately amplify 679 bp fragments. The specific primers (Table S1) from the *HsCBL8* cDNA were used to amplify 596 bp fragments, and *OsGAPDH* was used as reference gene (425 bp). The prepared 20 μL PCR reaction mix contained rTaq polymerase (Takara, Dalian China) (1.0 units), genomic DNA (20 ng), per primer (10 μM), and dNTP (10 mM). The following PCR program was used: 95°C for 4 min; 25 cycles of 94°C for 30 s, 58°C for 30 s, and 72°C for 60 s; and 72°C for 5 min. The PCR products were separated by 1.0% agarose gel electrophoresis.

### Southern blot analysis

Approximately 10 μg DNA per sample was separately and completely digested by *Hind* III and *Eco*R I, separately, and the restriction fragments were separated with 0.8% (w/v) agarose gel. Then, DNA was transferred onto a Hybond N^+^ nylon membrane (Boehringer, Mannheim, Germany) for 12 h, and the membrane was hybridized using a specific probe, including *HsCBL8* and an NOS sequence (Figure 1A). The probe (679 bp) was then amplified in accordance with primers (Table S1) using plasmid *pCAMBIA1300-35S-HsCBL8*. The probe was labeled with the enzyme horseradish peroxidase (Roche DIG High Prime DNA Labeling and Detection Starter kit II) for 10–12 h at 65°C. After hybridization, the membrane was washed twice with 2 × standard saline citrate (SSC) plus 0.1% sodium dodecyl sulfate (SDS) at 65°C for 10 min and washed twice with 0.5 × SSC plus 0.1% SDS for 10 min at room temperature. The membrane was covered with the detection reagent mixture for 30 min in accordance with the supplier's instructions. Finally, the membrane was wrapped in Saran wrap with 1 ml CSPD ready-to-use for 5 min and subsequently detected by a Fuji Photo Film Releases LAS-3000 Imaging System (Tokyo, Japan).

### GUS analysis

Histochemical staining of GUS activity was performed as follows. Plant tissues were incubated at 37°C overnight in GUS staining buffer (2 mM 5-bromo-4-chloro-3-indolyl-β-glucuronic acid in 50 mM sodium Pi buffer, pH 7.2) containing 0.1% Triton X-100, 2 mM K_4_Fe(CN)_6_, 2 mM K_3_Fe(CN)_6_, and 10 mM EDTA. The stained seedlings were sequentially transferred to 50, 70, and 100% (v/v) ethanol to remove chlorophyll. The stained materials were examined with a dissecting microscope (Leica MZ95) with a digital camera. GUS activity was also determined by fluorometric assay (Gallagher, 1992).

### Transgenic rice treated with high NaCL

The young rice seedlings (30 days) were treated with 125 mM NaCl in half-strength nutrient solution. Fresh weights were calculated at 0, 8, 16, and 24 h; plant phenotypes were photographed at 2, 4, and 6 days, and all leaves at 0, 24, and 72 h were used for analysis of proline, malondialdehyde (MDA), and Na^+^ and K^+^ contents, and rate of electrolyte leakage (REL). Plants without NaCl treatment were used as control. Meanwhile, proline content was determined as follows: 0.2 g fresh leaves were chopped and immersed in 5 ml of 3% sulphosalicylic acid at 100°C to extrac free proline. Proline content was then measured by UV Spectrophotometer (UV1000, LabTech) at 520 nm. MDA content was determined by the thiobarbituric acid (TBA) reaction. Leaves (0.2 g) were homogenized with 10 ml of 10% trichloroacetic acid (TCA) and centrifuged at 4000 rpm for 10 min. Part of the supernatant (2 ml) was mixed with 2 ml 0.6% TBA and incubated in 100°C water for 15 min, then cooled immediately on ice and centrifuged at 4000 rpm for 10 min. OD_450_, OD_532_, and OD_600_ were determined by UV spectrophotometry (UV1000, LabTech). MDA concentration (MDAC) were calculated through the formula; MDAC (μmol/L) = [6.45 (OD_532_ − OD_600_) − 0.56 OD_450_] × V × m, where V is the volume of the total extraction liquid and m is the sample weight. The leaves and roots were collected and dehydrated in a Muffle furnace at 500°C for 6 h. These ash samples were then extracted in 50% HNO_3_ overnight. The Na^+^ and K^+^ contents were measured by atomic absorption spectrophotometry (Z-5000 Polarized Zeeman, Hitachi, Japan). Membrane permeability can be reflected by the REL. Fresh leaves were collected and washed three times with deionized water to remove surface-adhered electrolytes. Each sample was divided equally and placed into two sealed vials containing 10 ml of deionized water. One vial was incubated at 25°C on a rotary shaker for 3 h, and then the electrical conductivity (EC1) was measured with a conductivity meter (162A, Thermo Orion, USA). Another vial was autoclaved at 100°C for 20 min, and the electrical conductivity (EC2) was determined by a conductivity meter. The REL can then be defined as REL = (EC1/EC2).

In these analyses, four biological replicates were performed independently, and four plants were used in per replicate.

### Real-time PCR analysis

XZ166 and CM72 plants (with three leaves, about 10 days) were treated with 200 mM NaCl, and the roots and shoots of three plants were separately collected at 0, 1, 6, 12, 24, and 48 h. Similarly, ZH11 and transgenic (*35S-HsCBL8*) seedlings (30 days) were treated with 125 mM NaCl, and shoot of three plants were separately collected at 0, 3, and 6 h. Three biological replicates were carried out independently. The primers adopted are listed in Table S1. *HvGapdh* and actin mRNAs were separately employed as internal controls for barley and rice. Real-time PCR was performed using the CFX96 system (Bio-Rad, America) with iTaq Universal SYBR® Green Supermix (Bio-Rad). The PCR program was implemented as follows: 95°C for 3 min and 40 cycles of 95°C for 15 s, 58°C for 15 s, and 72°C for 15 s.

### Statistical analysis

Statistical analysis for real-time PCR was conducted using the software CFX Manager (version 2.1), and the relative expression level of per gene was calculated by the 2^−ΔΔCT^ method (Livak and Schmittgen, 2001). Moreover, student's *t*-test was applied for each parameter studied. Differences were considered significant at *P* <0.05 and highly significant at *P* <0.01.

## Results

### Cloning of *HsCBL8*

**Figure 2:**
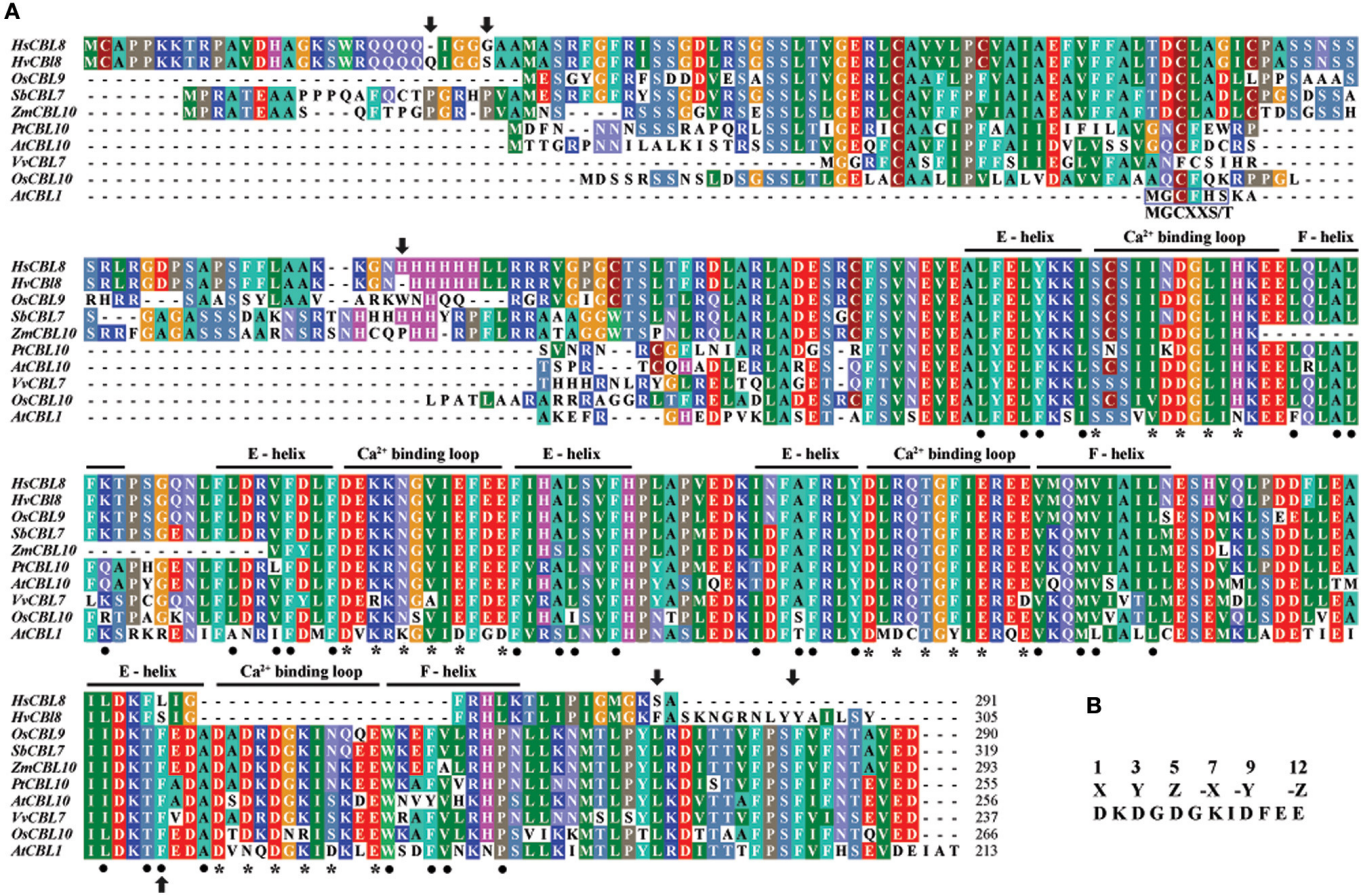
**Analysis of the CBL amino-acid sequences. (A)** Alignment of the amino-acid sequences belonging to the sub-cluster (II-I-I-II) of some species, including HsCBL8, HvCBL8, OsCBL9, SbCBL7, ZmCBL10, PtCBL10, AtCBL10, VvCBL7, and OsCBL10. AtCBL1 was used as the control. The four EF–hand calcium-binding motifs are clearly indicated. Asterisks and dots denote the amino-acid residues important for calcium binding and the EF–hand structure, respectively. Arrows show the amino acids altered between HsCBL8 and HvCBL8. Conserved myristoylation motifs (MGCXXS/T) in the N-termini of AtCBL1 was marked by a blue box. **(B)** EF–hand consensus sequence in accordance with Kolukisaoglu et al. (2004).

The first 494 bp cDNA sequence amplified with degenerate PCR primers (Table S1) was found similar to the full-length cDNA sequence of the gene FLbaf27i23 in barley (*H. vulgare subsp. vulgare*) (http://www.shigen.nig.ac.jp/barley/). BLASTX (http://blast.ncbi.nlm.nih.gov/Blast.cgi) analysis of the coding sequence (CDS) predicted 915 nucleotides, which code HvCBL8 with 305 amino acids (Figure 2A). A fragment of 873 bp at the CDS was cloned from wild barley XZ166 using primers based on FLbaf27i23, which encodes the HsCBL8 protein of 291 amino acids (Figure 2A, NCBI, AEM44693.1). We performed IPCR of the 2388 bp fragment (NCBI ID, HQ696007.1) upstream the initial codon. Moreover, 31 *cis* elements of the upstream 1000 bp from initiation codon were predicted by PlantCARE. Ten of these elements were light response elements, five were MeJA response elements, four were ABA response elements, four were endosperm expression elements, three were drought response elements, two were GA-responsive and circadian control elements, and one was a salicylic acid (SA) response element (Table 1). The predicted *cis* elements of the *HsCBL8* promoter sequence indicated that *HsCBL8* could be induced by different stresses (Table 1).

**Table 1:** **Prediction of ***cis***-elements responding to environment stimuli in ***HsCBL8*** promoter (the upstream 1000 bp from the initiation codon) using software PlantCARE**.

***Cis*-element name**	**DNA sequence (5′–3′)**		**Number of *cis*-elements**	**Function**
ABRE	CACGTG/CCTACGTGGC/CCGCGTAGGC/TACGTG		4	Abscisic acid responsiveness
ACE	ACGTGGA	1	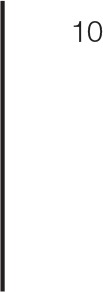 10	Light responsive element
Box 4	ATTAAT	1		
G-box	CACGTG/CACGTA/CACATGG/CACGTC/TACGTG	5		
GATA	GATAGGG	1		
GT1	GGTTAAT	1		
I-box	GATAGGG	1		
CGTCA/TGACG	CGTCA/TGACG	5		MeJA-responsiveness
GARE	AAACAGA	2		Gibberellin-responsive element
MBS	TAACTG/CAACTG/TAACTG	3		Drought-inducibility
Skn-1	GTCAT	4		Endosperm expression
TCA	GAGAAGAATA	1		Salicylic acid responsiveness
Circadian	CAANNNNATC	2		Circadian control

### Phylogenetic analysis of *HsCBL8*

**Figure 3:**
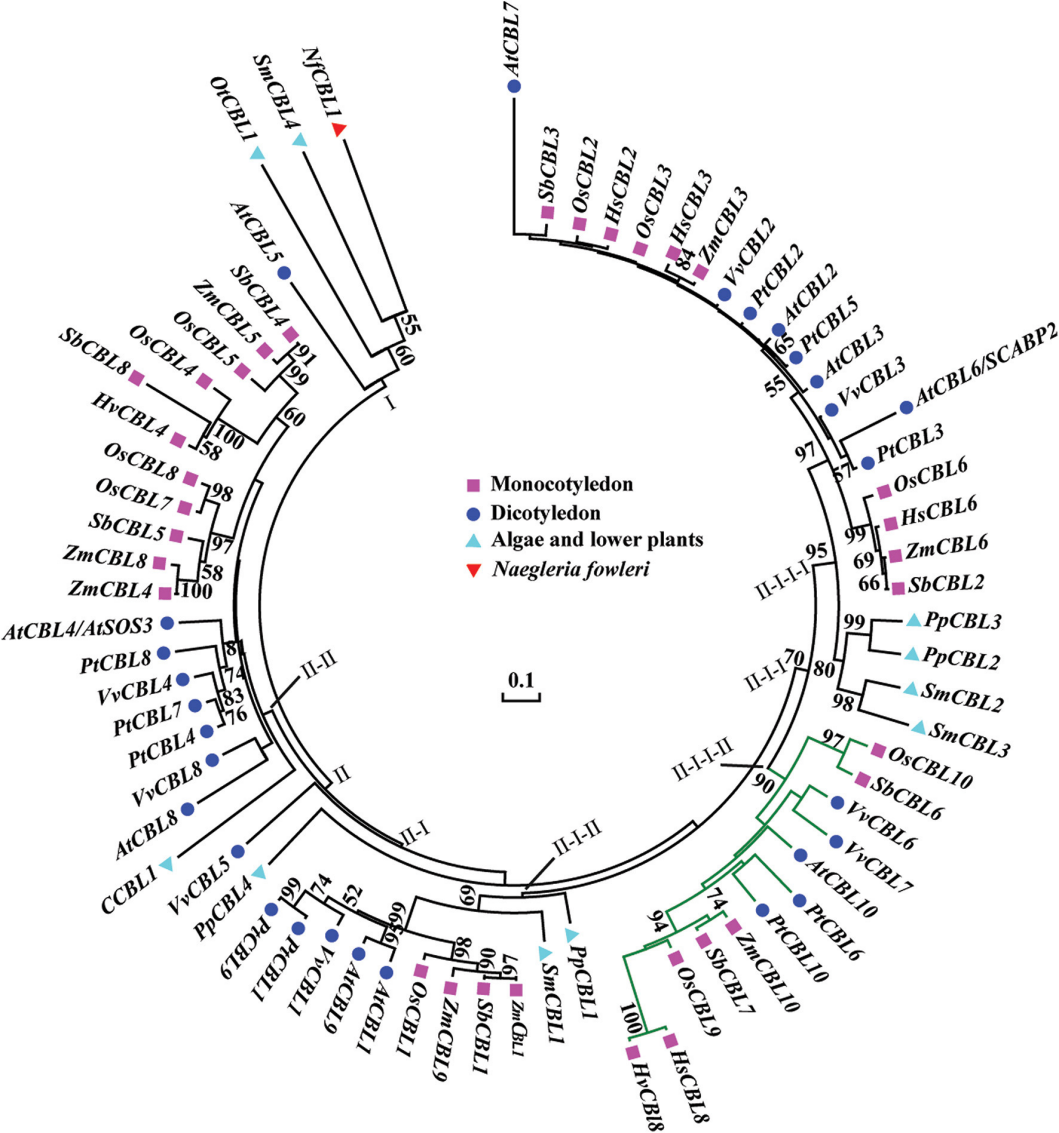
**Phylogenetic relationships of CBL proteins from algae, lower plants, higher plants, and protozoan ***Naegleria fowleri*** (Nf)**. The NCBI accession numbers of the CBL proteins in this paper are presented in Table S2. The bar shows the evolutionary distance, and the number at the nodes represents the reliability percentage (%; only >50% is shown) of the bootstrap values based on 1000 replications. Codification: Hs, *Hordeum spontaneum*; Hv, *H. vulbare*; Os, *Oryza sativa*; Zm, *Zea mays*; Sb, *Sorghum bicolor*; At, *Arabidopsis thaliana*; Pt, *Populus trichocarpa*; Vv, *Vitis vinifera*; Ot, *Ostreococcus tauri*; C, *Chlorella* sp.; Pp, *Physcomitrella patens*; Sm, *Selaginella moellendorfii*.

To study the phylogenetic relationship of *HsCBL8*, the amino acid sequence of HsCBL8 was compared with 71 CBL genes from plant species including monocot (rice, sorghum, maize and barley), dicot (*Arabidopsis*, grape and populous), and protozoan (*Naegleria fowleri)*. The corresponding IDs are presented in Table S2. Accordingly, members of the family CBL family could be clustered into two groups (Figure 3). Group I only comprised four members. Three belonged to simple plants and *N. fowleri*, and one represented *Arabidopsis* (*AtCBL5)*. By contrast, Group II was divided into two sub-clusters: II-I and II–II. Moreover, II-I was grouped into II-I-I and II-I-II, whereas II-I-I was sub-clustered into II-I-I-I and II-I-I-II. *HsCBL8* was found closely related to rice *OsCBL9* (63.70%) and thus placed in the group II-I-I-II, which included 12 CBLs (Figure 3). Results revealed that most of CBLs belonging to monocots were slightly deviant phylogenetically from dicot species, whereas the CBLs of lower plants were found in close relation to higher plant species in each group (Figure 3). Moreover, HsCBL8 was significantly different to HvCBL8 in terms of a 14-amino-acids deletion at its nitrite end, and five other changed sites, including three changed, one deletion, and one more amino acid (Figure 2A). Normally, the CBL protein contains four EF–hand domains for Ca^2+^ ion binding (Figure 2B), but the last domain was lost in HsCBL8 and HvCBL8 (Figure 2A). Obviously, a conserved myristoylation motif (MGCXXS/T) in the N-terminus of AtCBL1 was not discovered in the group II-I-I-II (Figure 2), including HsCBL8 (Figure 2A).

### Expression analysis of *HsCBL8* in barley under salt stress

**Figure 4:**
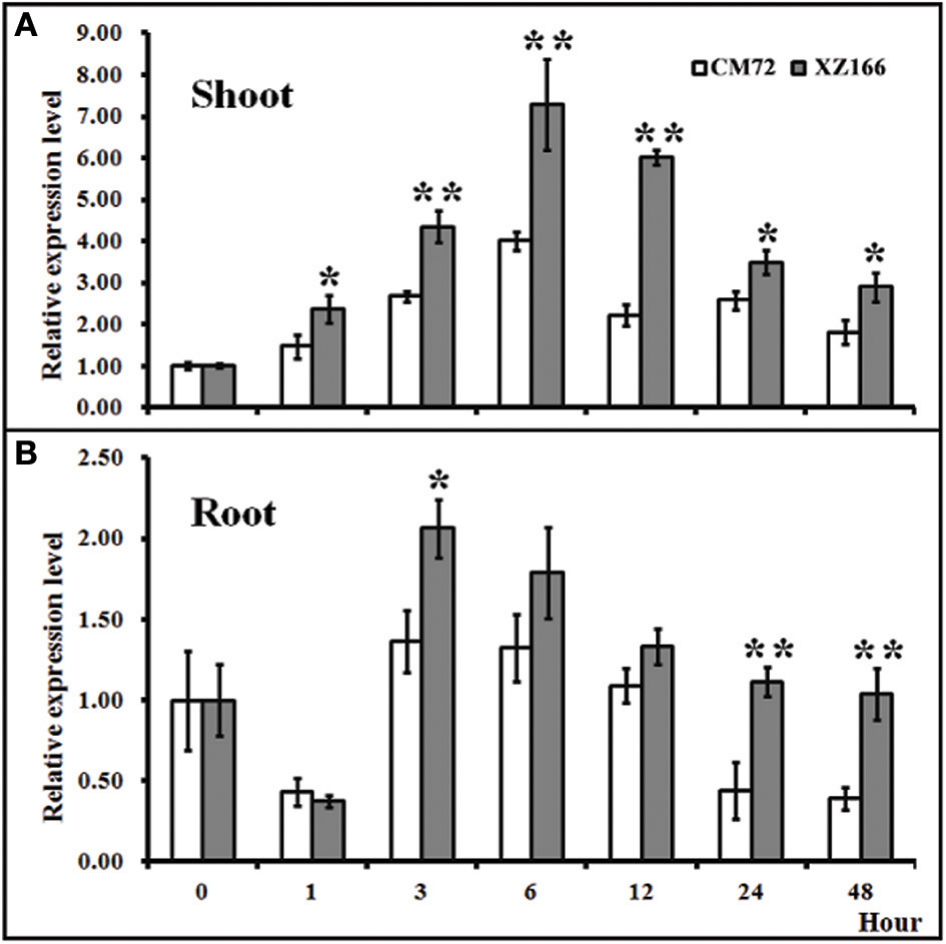
**Relative expression levels of ***CBL8*** between XZ166 and CM72 plants treated with 200 mM NaCl. (A)** Shoot, and **(B)** root. The expression level for 0 h treatment was set to 1. Values include the means of three independent replicates. *Indicates a statistically significant difference at *p* <0.05 and **signifies a highly significant difference compared with those of the CM72.

To evaluate whether *HsCBL8* in XZ166 and *HvCBL8* in CM72 could respond to salt, we conducted real time reverse-transcription PCR analysis to test their expression patterns in the control and under salt stress. Results revealed that the expression of both the genes in their respective plant species, were significantly induced in their shoots and roots, respectively, in response to 200 mM Nacl stress (Figure 4). Overall, *HsCBL8* was expressed to significantly higher levels in both shoots and roots than that in *HvCBL8*. The expression profile of both the genes in the shoot generally followed a sigmoid curve in general. The expression level was enhanced in the first hour of NaCl application, reaching its peak after 6 h and then gradually declining with prolonged salt stress from 12 to 48 h, respectively (Figure 4A). In the roots, the expression of the *CBL8s* in both XZ166 and CM72 was greatly suppressed (Figure 4B) during first hour of NaCl treatment. However, both the genes were highly expressed at 3 and 6 h of stress treatment. Afterward, a gradual decline was observed in the expression pattern after up to 48 h of stress treatment. However, significant differences in transcriptional levels were observed between *HvCBL8* and *HsCBL8* at 3, 24, and 48 h in the roots (Figure 4B), revealing the variation in the degree of sensitivity of these genes to salt stress.

### *HsCBL8* transgenic rice and its response to salt stress

**Figure 5:**
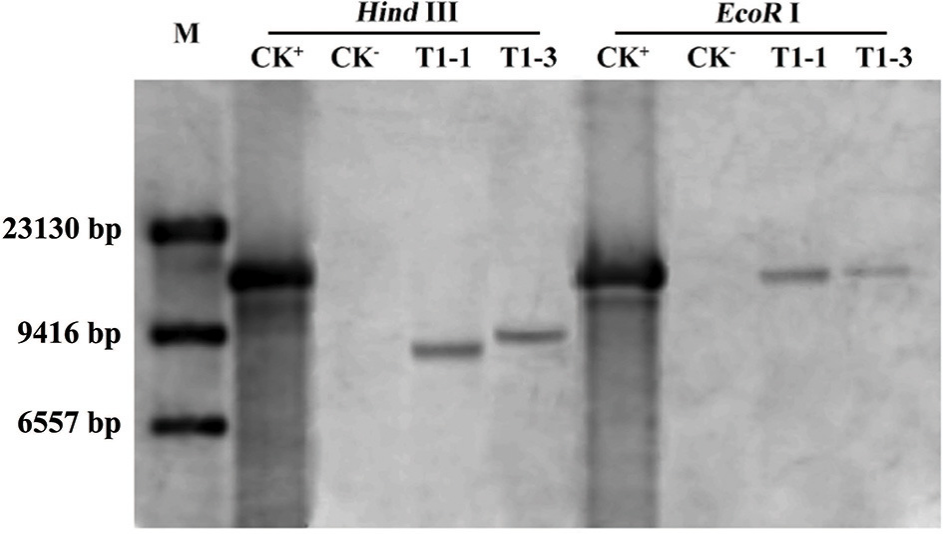
**Southern blot analysis of T1 transgenic rice plants**. M, DNA marker (λ-DNA/*Hind* III); CK^+^, *pCAMBIA1300-35S-HsCBL8* (12.9 kbp); CK^−^, ZH11; T1-1 and T1-3 selected transgenic lines with one copy of *HsCBL8* integrated into the rice genome.

To study the role of *HsCBL8* in rice, an *Agrobacterium* strain *EHA105* harboring the plasmid *pCAMBIA1300-35S-HsCBL8*, was transformed into rice ZH11 using embryo calli induced from mature seeds. A Southern blot analysis using two restriction enzymes, *Hind* III and *Eco*R I, revealed the presence of two copies (T1-1 and T1-3) of the T1 transgenic line of rice (Figure 5). Of these transgenic plants, 20 T2 seedlings of T1-1 and 20 T2 seedlings T1-3 were verified by PCR and resulted in the detection of 9 homozygous transgene (*35S-HsCBL8*) plants with 679 bp band length. Furthermore, T3 seeds from T2 homozygous lines namely T2-1-3 (L1) and T2-3-9 (L2) (Figure S1), were selected for further analysis to evaluate the functional attributes of the gene for its tissue-specific expression.

**Figure 6:**
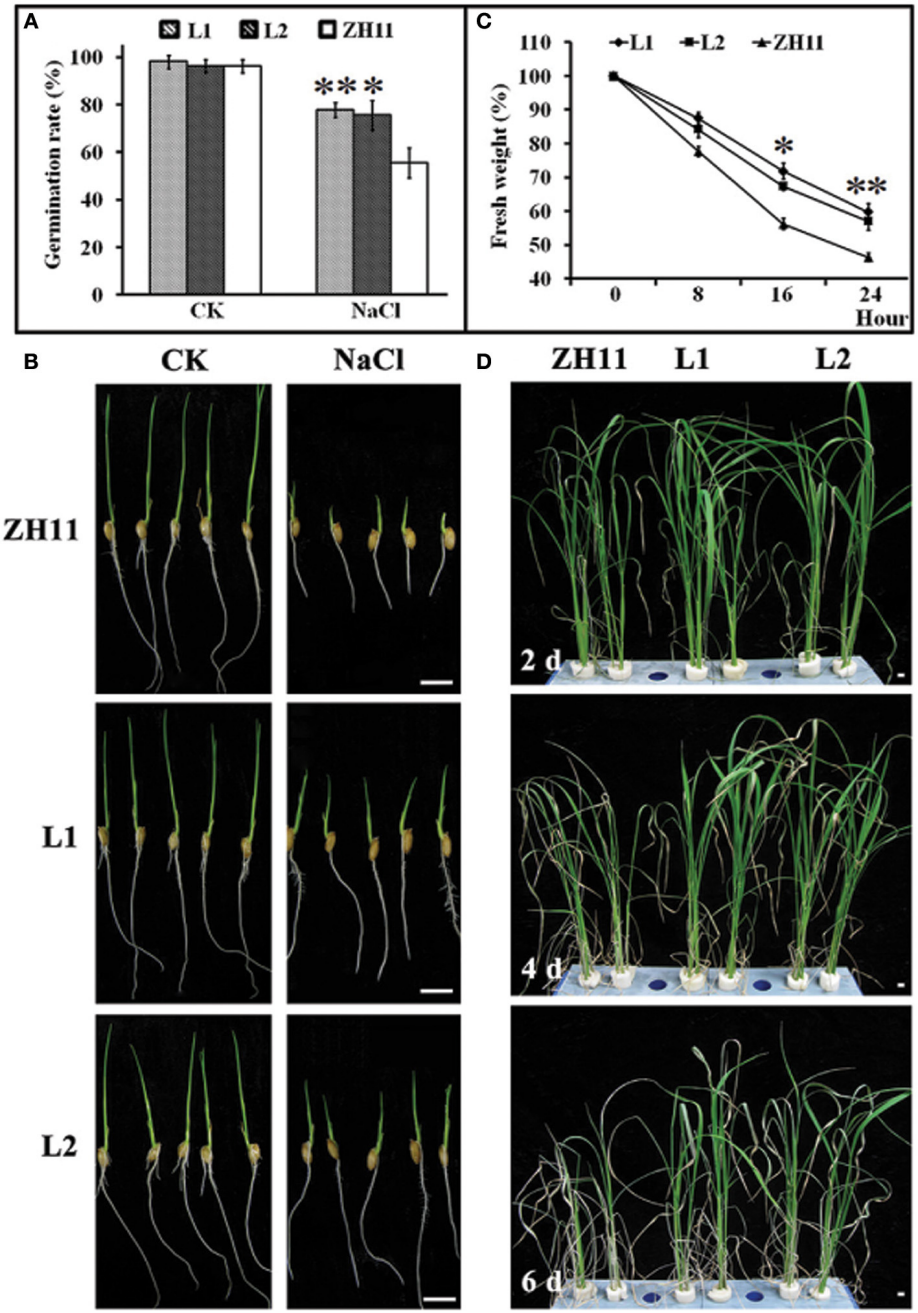
**Phenotypic changes of transgenic rice lines induced by salt stress. (A)** Germination rate of seeds treated with 125 mM NaCl for 5 days, and morphological characteristics of rice young seedlings **(B). (C)** Water loss, 2 week pre-cultured plants were treated with 125 mM NaCl. **(D)** Photos of rice shoots with treatment of 125 mM NaCl, the seedlings were pre-cultured for 30 days in normal conditions. Values include the means ± standard deviation (SD) (*n* = 3). *(*p* <0.05) and **(*p* <0.01) indicate that these values were statistically significantly and highly significantly different respectively, compared with that of the ZH11. Bar = 1 cm. CK, normal condition; L1 and L2, transgenic lines.

To evaluate the possible role of *HsCBL8* in salt tolerance, the seedling growth and morphology of the transgenic rice lines (L1 and L2) were compared with that of the control (ZH11) plants (Figure 6). The comparison was conducted under normal and 125 mM NaCl stress conditions. We found insignificant differences in seed germination (Figure 6A) and seedling fresh weight (Figure 6C) between the transgenic and ZH11 plants under the normal growth conditions as evident from the seedling growth (Figure 6B). By contrast, fairly obvious and significant variations in seed germination rate as well as seedling fresh weight were observed between the transgenic lines and non-transgenic ZH11 under 125 mM NaCl stress (Figures 6A,C). The overall germination rate of both transgenic and ZH11 seeds were reduced under salt stress relative to that of the untreated seeds. However, the lines L1 and L2 thrived best at around 75% germination which was 20% higher than that of ZH11 (Figure 6A). This finding was evident from the stunted growth of the radicles and plumules of the ZH11 seedlings under salt stress (Figure 6B). Moreover, the rate of water loss from the transgenic lines was significantly lower than that of ZH11 when the application of salt stress was prolonged from 0 to 24 h. Obvious differences between the fresh weights of seedlings of transgenic lines and ZH11 were observed at 16 (*p* <0.05) and 24 h (*p* <0.01) of salt stress (Figure 6C). Furthermore, we tested salt tolerance in the plants at the well-grown vegetative stage. We then planted the seedlings of L1, L2 and ZH11 for 30 days in normal conditions hydroponically. After 30 days of growth, we applied 125 mM NaCl stress, initially for 2 days, 4 days, and finally 6 days (Figure 6D). The affects of NaCl treatment were significantly prominent on the leaves of the ZH11 plants, especially on the older leaves. The degree of severity of the effect was increased with the exposure time of the ZH11 plants to the treatment from 2 to 6 days compared with that in the transgenic lines. This effect was manifested as withering of older leaves, increased water loss, and curling of young leaves in the ZH11 plants compared with those in the L1 and L2 plants. The transgenic plants showed better performance as exhibited by the strong vitality of the top new leaves, even after 6 days of NaCl treatment (Figure 6D). These results indicate the obvious role of *HsCBL8* in salt tolerance.

### Role of *HsCBL8* in the salt stress response in transgenic rice

**Figure 7:**
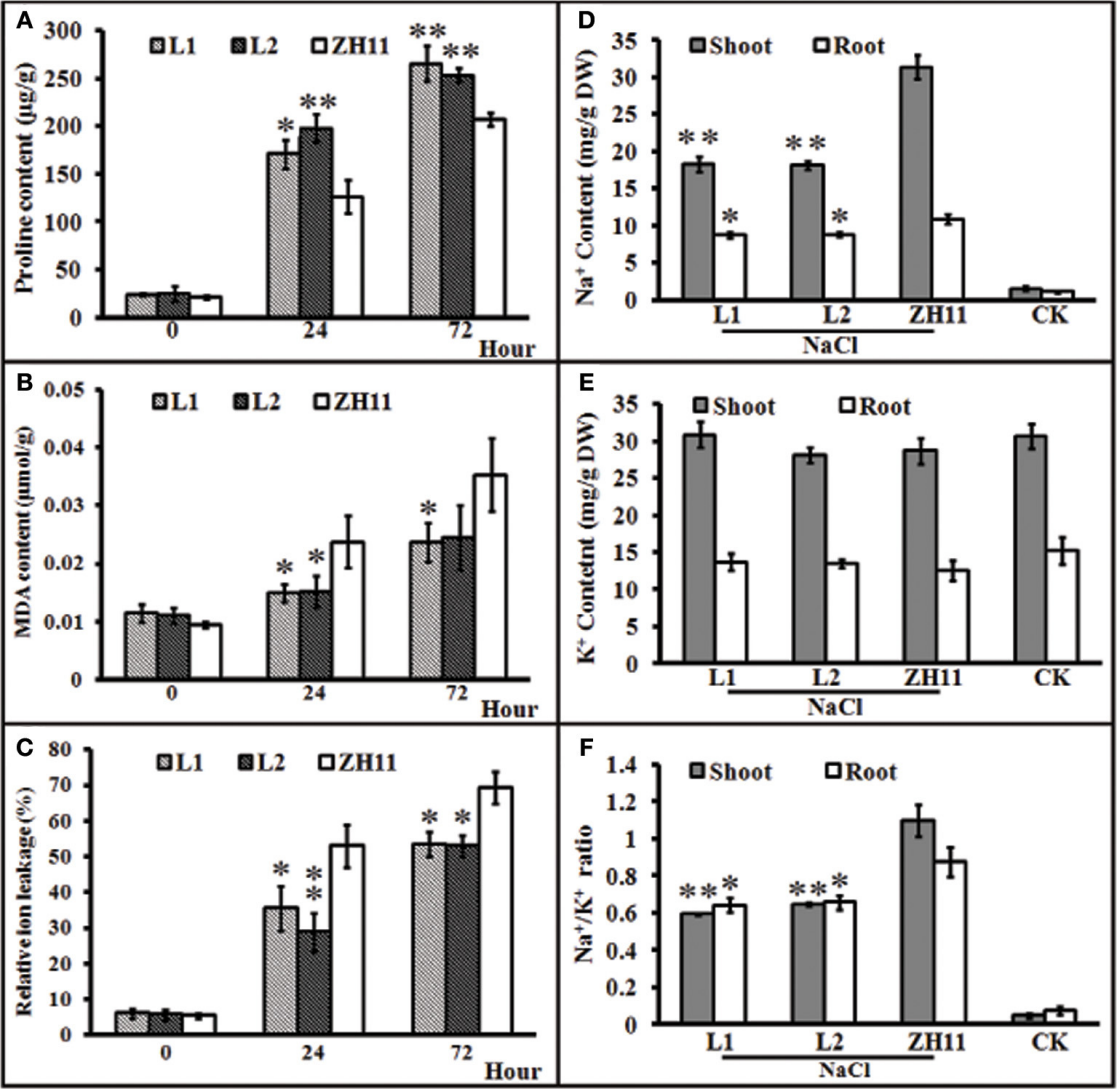
**Characteristics of transgenic rice lines and ZH11 treated with salt stresses. (A–C)**, physiological indexes reflecting salt tolerance: **(A)** proline content, **(B)** MDA content, and **(C)** relative ion leakage. Plants pre-cultured for 2 weeks were treated with 125 mM NaCl. **(D–F)**, Na^+^ and K^+^ contents (mg/g DW) of shoots and roots of transgenic and ZH11 plants. **(D)** Na^+^ and **(E)** K^+^ levels and **(F)** Na^+^/K^+^ ratio. Na^+^ and K^+^ contents were measured after 6 days of 125 mM NaCl treatment. The treated plants were pre-cultured for 30 days. Values include the means ± SD (*n* = 4). *(*p* <0.05) and **(*p* <0.01) indicate that these values were statistically significantly and highly significantly different, respectively, compared with that of ZH11 or CK. CK and ZH11 growing in normal conditions; L1 and L2, transgenic rice lines.

Generally, the accumulation of compatible osmolytes such as proline, are associated to the stress tolerance of the corresponding plant (Ahmed et al., 2013; Mekawy et al., 2015). Meanwhile, quantification of MDA concentrations and REL is considered as an efficient indicator of the structural integrity of the plant membranes in response to abiotic factors, such as salt stress. To evaluate the functional attributes of overly expressed *HsCBL8* for rice tolerance against salt stress, we analyzed the proline and MDA concentrations, and rate of REL. Accordingly, the *HsCBL8* overexpression in the transgenic rice resulted in the accumulation of 35 and 56% additional proline content in the L1 and L2 lines, respectively, after 24 h of salt stress compared with that in ZH11 (Figure 7A). Moreover, plant exposure to plants to salt stress for 72 h resulted in an overall increase in proline content in both the transgenic and ZH11 plants compared with those in 24 h. Even so, 33% and 25% accumulation of additional proline content was observed in L1 and L2 respectively, relative to that in ZH11. This result indicates the possible role of *HsCBL8* in salt tolerance. On the contrary, *HsCBL8* overexpression significantly reduced the MDA content and REL in the transgenic lines compared with the non-transgenic ZH11 plants under salt stress. This reduction in MDA content was in the order of 36% (in both transgenic lines) at 24 h, but 31 and 29% in L1 and L2, respectively, at 72 h of salt stress compared with the contents in ZH11 (Figure 7B). Similarly, 33 and 46% reduction in REL was observed in L1 and L2 respectively, at 24 h of salt treatment, whereas, reduction of 23% and 24% reduction in REL was noted in L1 and L2, respectively, at 72 h of salt stress compared with that in the ZH11 plants (Figure 7C).

### Na^+^ and k^+^ contents in the *HsCBL8* transgenic rice

Generally, the ability of a crop variety to inhibit the uptake of potentially toxic ions such as Na^+^ and preferential uptake of K^+^ to keep the balance between ions is regarded as a desirable trait for salt tolerance. To investigate whether *HsCBL8* could induce the salt tolerance trait in transgenic rice, we analyzed the status of Na^+^ and K^+^ ions and their comparative ratio in roots and shoots under control, as well as salt stress conditions. Plants of both transgenic as well as ZH11 pre-grown for 30 days under normal condition were exposed to 125 mM NaCl stress for 6 days. After 6 days, analysis of the ions revealed insignificant differences in Na^+^ and K^+^ concentration among unstressed transgenic lines and ZH11. By contrast, under salt stress, Na^+^ accumulation was generally noted at higher content in the shoots than in the roots in either of the transgenic lines and ZH11 plants. However, the Na^+^ contents in ZH11 plants were significantly higher in the shoots (42%) as well as the roots (18.5%) than in both of the transgenic lines (Figure 7D). On the other hand, salt stress induced a slight change in root and shoot K^+^ contents between the transgenic lines and ZH11 plants applied with or without NaCl treatment (Figure 7E). Consequently, the Na^+^/K^+^ ratio in both shoot and root was lower in transgenic lines than that in ZH11 plants (Figure 7F), indicating that the overexpression of *HsCBL8* in rice could regulate and induce salt tolerance.

### Expression levels of some stress responding genes in the transgenic rice harboring *HsCBL8*

**Figure 8:**
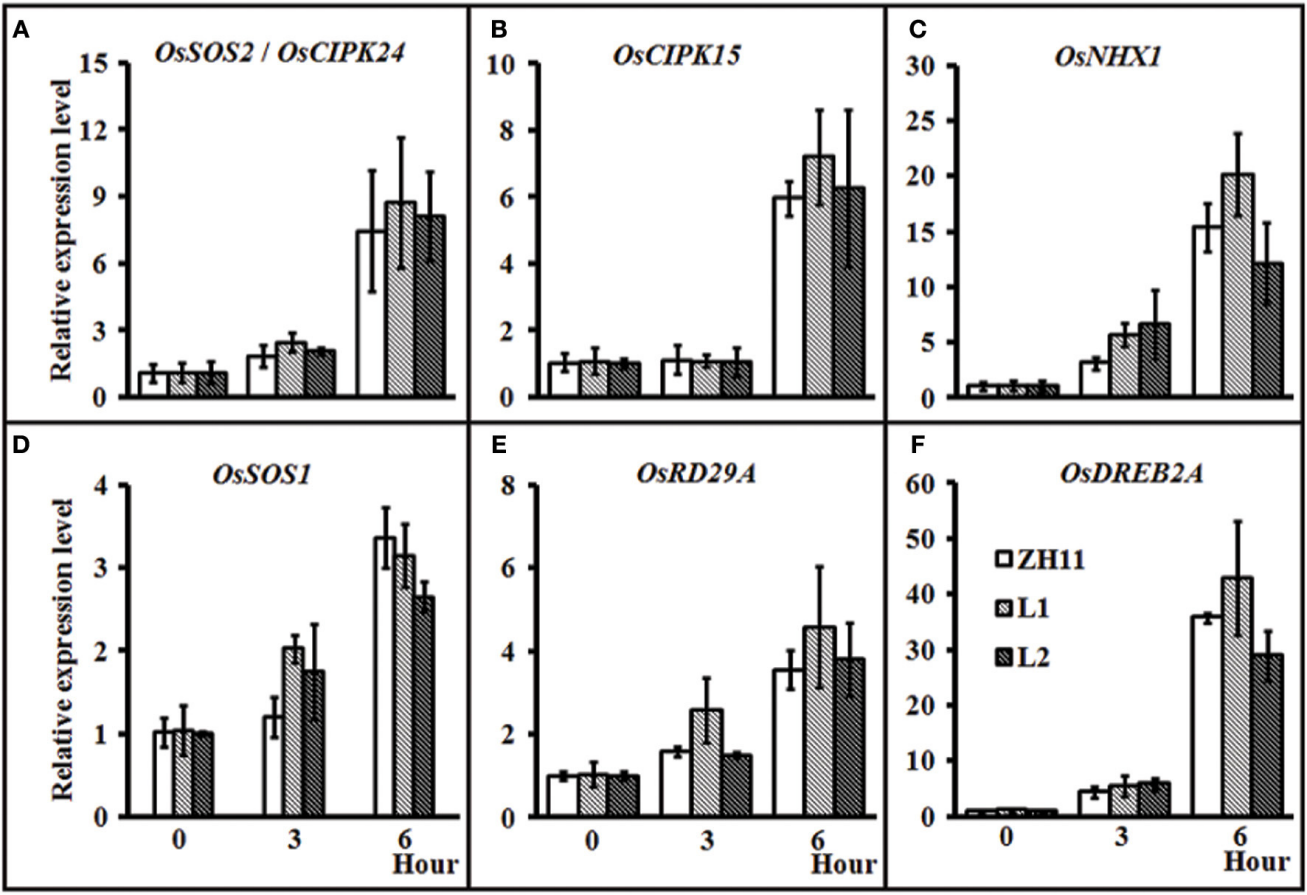
**Relative expression of some stress-responsive genes in rice ZH11 and two transgenic lines with treatment of 125 mM NaCl for 0, 3, and 6 h**. The plants were pre-cultured for 3 weeks in hydroponic solution. The expression level of each gene for WT (0 h) was set to 1. Values include the means ± SD (*n* = 3). *Actin1* (NCBI ID: NC_008396) was used as reference gene. NCBI ID: *OsSOS2* **(A)**, DQ248963; *OsCIPK15* **(B)**, AB264037; *OsNHX1* **(C)**, AB021878; *OsSOS1* **(D)**, AY785147; *OsDREB2A* **(E)**, JQ341059, and *OsRD29A* **(F)** (Rao et al., 2011).

To investigate whether *HsCBL8* could regulate the salt-responsive genes in rice, we performed the gene expression profiling of the *35S-HsCBL8* transgenic lines (L1 and L2) in comparison with the non-transgenic plants under normal conditions and salt stress. The genes selected were either specifically salt responsive (*OsSOS*2/*OsCIPK24, OsNHX1*, and *OsSOS1*) and/or responsive to multiple stresses (abiotic or biotic) (*OsCIPK15, OsRD29A*, and *OsDREB2A*). Under normal growth conditions (unstressed, 0 h), we observed extremely weak expression, as well as insignificant variations in the transcriptional levels of all the selected genes in both ZH11 and transgenic plants. This result indicates that *HsCBL8* exerted an almost negligible interaction with these genes (Figures 8A–F) in the unstressed environment. At the onset of stress application (125 mM NaCl) initially for 3 h, the overall relative expression level of the genes was enhanced relative to that in the unstressed plants. However, the difference between the transgenic lines and ZH11 was insignificant. Moreover, duration of salt stress exposure was prolonged to 6 h, all the genes were found substantially up-regulated compared with the unstressed plants (0 h) or those subjected to salt stress for 3 h. However, a a comparison between the transgenic lines and ZH11 at 6 h of salt stress revealed that the overexpression of *HsCBL8* in both the transgenic rice lines up-regulated some of the genes, such as *OsSOS*2/*OsCIPK24, OsCIPK15*, and *OsRD29A*, to a higher level than that in ZH11 (Figures 8A,B,E). Even so, the overall variation was insignificant, similar to the one observed in plants exposed to 3 h of salt stress or the untreated plants. These results indicate that the overexpression of *HsCBL8* in rice could not affect the regulation of salt-responsive genes significantly, showing the negligible interaction of *HsCBL8* with all the selected genes expressed in response to salt stress.

### GUS activity driven by the *HsCBL8* promoter in response to abiotic stress in transgenic *arabidopsis*

**Figure 9:**
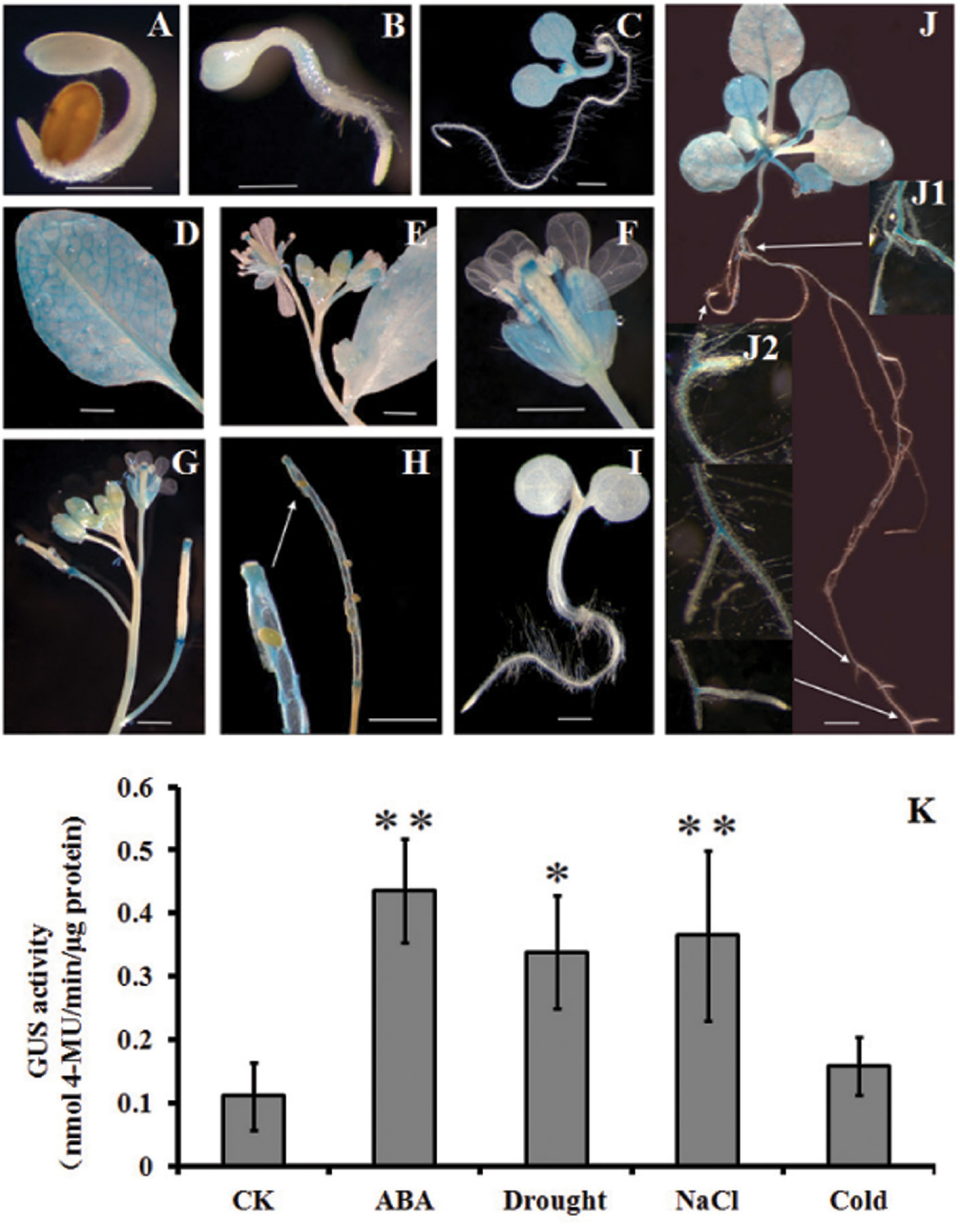
**Histochemical analysis of GUS activity in transgenic ***Arabidopsis*** plants. (A–J)** GUS staining at different developmental stages; **(K)**, GUS activity responding to ABA, drought, NaCl, and cold treatments. **(A)** 2-day-old seedlings, **(B)** 3-day-old seedling, **(C)** 6-day-old seedling, **(D)** rosette leaf, **(E,F)** flowers, **(G)** young siliques, **(H)** mature siliques, **(I)** *Col-0*, and **(J)** 2-week-old seedlings. **(K)** 4-week-old seedlings supplemented with 100 μM ABA, 20% PEG6000, and 100 mM NaCl. For cold treatment, the seedlings were transferred to a 4°C growth chamber in the dark. GUS activity was assayed after 12 h treatment. The values are expressed as means ± SD (*n* = 4). *Indicates statistically significant difference at (*p* <0.05) and **indicates a highly statistically significant difference at (*p* <0.01) compared with CK. Bar = 5 mm. The line with arrow shows the magnified tissue portion.

To have more detailed perspective of expression pattern of *HsCBL8* in response to various abiotic stresses, the promoter fragment of *HsCBL8* was fused with the GUS reporter gene and transferred to *Arabidopsis* to develop *HsCBL8*_*Pro*_*-GUS* transgenic lines (Figure 1B). The expression of GUS activity was examined in 17 independent transgenic lines. Except for three lines (with zero GUS activity), the other 14 lines exhibited similar tissue-specific expression pattern. However, the intensity of the colors varied among lines, probably because of the position effect. The GUS expression pattern of a representative line (TGUS-7) is presented herein (Figures 9A–J). Under normal conditions, GUS signals were extremely weak in the cotyledon apex at the second day after germination (Figure 9A), whereas, the hypocotyl stele was stained light blue at the third day (Figure 9B), showing the activity of GUS promoted in hypocotyl and cotyledons (Figure 9C) and further strengthened in the vascular bundles of elder rosette leaves (Figures 9D,J). However, no activity was observed in the newly formed root tissues, including lateral root tips (Figures 9D,J), instead the blue stain was observed within the root stele tissue (Figure 9J) and the junction tissue of tap and lateral roots (Figure 9J). Furthermore, GUS activity at the reproductive phase was detected in sepals, filaments, and tissues between the style and stigma, and tissues between the silique and silique petiole but no GUS signal was observed in the petals and seeds (Figures 9E–H).

Some *cis* elements in the *HsCBL8* promoter sequence predicted by PlantCARE, such as ABRE and MBS (Table 1), normally respond to ABA signal and drought stress, respectively. To evaluate the response of the *HsCBL8* gene to ABA, drought, NaCl and cold stress, GUS activity was measured by fluorometric assay (Figure 9K). Four-week-old *Arabidopsis* seedlings were supplemented with 100 μM ABA, 20% PEG6000, and 100 mM NaCl to apply ABA, drought and salt stress, respectively. For cold treatment, the seedlings were transferred to a growth chamber under dark conditions at 4°C. GUS activity was assayed after 12 h treatment. Accordingly, compared with untreated plants, GUS activity was observed to significantly (*p* <0.01) higher levels in the plants treated with ABA (294%), NaCl (230%), and PEG6000 (*p* <0.05; 205%), respectively, whereas, an increase of only 43% was obtained with cold treatment, indicating the possible response of *HsCBL8* to these stresses.

## Discussion

*Hordeum spontanum*, derived from Qinghai-Tibet Plateau (origin center of the cultivated barley) is known as a highly salt-tolerant species (Dai et al., 2012; Wu et al., 2013). Being a member of same crop family, studies on the elite genes of this species in rice could help us better understand of the salt-tolerance mechanisms in wild barley. To obtain insight about the salt stress responses in rice related to elite gene(s) from wild barley, we selected the *HsCBL8* gene and thoroughly analyzed its functional relations with other genes in response to the salt stress regime. Initially, we conducted the phylogenetic analysis of *HsCBL8* and revealed that the encoded protein belongs to the group of CBL proteins only modified with N-myristoylation or S-acylation (Batistic et al., 2012), (Figure 2A). Thus, these proteins are involved in close phylogenetic relationships with the members of group II-I-I-II (Figure 2) besides 10 other CBL proteins. This group was clustered into a clade defined as a transmembrane (TM) helix (Kleist et al., 2014) in accordance with AtCBL10, which harbors a unique transmembrane-spanning region (Kim et al., 2007). Moreover, ZmCBL10, OsCBL9, SbCBL7, and HsCBL8 also harbor long N-terminal extensions (Figure 2A), indicating that the long N-terminal amino-acid sequence of HsCBL8 may be involved in sub-cellular localization. However, we could not find the transmembrane domain in the HsCBL8 protein sequence similar to that in AtCBL10. Therefore, further investigation must to be conducted to confirm the assumption.

In general, each CBL protein harbors four EF–hand motifs (Figures 2A,B), but not all of the CBL proteins contain four canonical EF–hand motifs. For instance, five CBL proteins in *Arabidopsis* (CBL2, CBL3, CBL4, CBL5, and CBL8) do not possess any canonical EF hand, whereas, CBL6, CBL7 and CBL10 contain one and CBL1 and CBL9 contain two canonical EF hands (Batistic et al., 2012). Herein, both HsCBL8 and HvCBL8 lack the fourth EF hands (Figure 2A). Moreover, the structural and numeral differences in the EF hands demonstrate variations in Ca^2+^ binding affinity and interactions between the CBL and CIPK proteins. For example, crystal structure analyses of AtCBL2 and AtCBL4 revealed that AtCBL2 bound only to CIPK14 with four Ca^2+^ ions in all four EF hands (Akaboshi et al., 2008), whereas, AtCBL4 bound only to CIPK24 with two Ca^2+^ ions in two EF hands (Sánchez-Barrena et al., 2007). Therefore, the model representing the cooperative binding of the EF hand and Ca^2+^ ion in HsCBL8 could not be deduced from the current results. This finding suggests that some known downstream factors of CBLs were not regulated by *HsCBL8* (Figure 9). Instead *HsCBL8* might interact with other factors to regulate downstream genes responding to salt stress in transgenic rice.

To verify this hypothesis, we investigated the growth performance of transgenic rice lines with *HsCBL8* expression in response to salt stress. We observed that the constitutive expression of *HsCBL8* significantly improved the tolerance against salt stress in rice. Germination rate was the first index that showed the obvious role of *HsCBL8* against salt stress in the transgenic rice treated with 125 mM NaCl (Figure 6A). These findings followed the similar trends reported for other *CBLs* in various plant species, such as the transgenic *Arabidopsis* harboring *35S-AtCBL5* (Cheong et al., 2010) and the poplar containing *35S-PeCBL10* (Li D. D. et al., 2013). Moreover, scholars reported that *OsCBL2* may be involved in a GA-signaling pathway that leads to the vacuolation of the aleurone cell (Hwang et al., 2005). Meanwhile, the attributes of *AtCBL1* in terms of positive response to gibberellins (Li Z.-Y. et al., 2013) and negative response to ABA (Pandey et al., 2008) during seed germination and seedling development could provide evidence supporting the similar characteristics of *HsCBL8* under the salt stress condition. The overexpression of *HsCBL8* in our study also decreased Na^+^ uptake partly in rice (Figures 7D–F). Moreover, *AtCBL10*, which shares the same cluster with *HsCBL8* (Figure 3), responded similarly to salt stress by modulating the activities of Na^+^ transporters (Kim et al., 2007; Quan et al., 2007). *AtCBL10*'s homologs *PeCBL10* (Li D. D. et al., 2013) and *NsylCBL10* (Dong et al., 2015) in roots, whereas, *PtCBL10A* and *PtCBL10B* (Tang et al., 2014) in shoots also showed salt tolerance by regulating Na^+^ balance, indicating that *HsCBL8* might regulate some Na^+^ transporters or channels involved in the efflux of excess Na^+^ under salt stress. However, K^+^ uptake was slightly affected in the transgenic rice with *HsCBL8* (Figure 7E). Recent evidence showed that *AtCBL10* may directly compete with *CIPK23* for binding to AKT1 and negatively modulate AKT1 activity (Ren et al., 2013) but AtCBL1/9-CIPK23-AKT1 (K^+^ transporter) might enhance K^+^ uptake under low-K^+^ conditions (Xu et al., 2006; Li et al., 2014). These findings suggested that *HsCBL8* may lessen the antagonism of high Na^+^ to K^+^ uptake. Nevertheless, the underlying mechanism remains unknown. Unexpectedly, the transcriptional levels of some known downstream factors of *CBLs* in transgenic rice were not regulated by *HsCBL8* (Figure 8), including the Na^+^ transporters *OsNHX1* and *OsSOS1*. Thus, *HsCBL8* might interact with other known or unknown factors for improving salt tolerance, membrane protection (Figures 7B,C), promoting proline accumulation (Figure 7A), and so on.

To confirm the functional attributes of *HsCBL8* in plant growth and development, we conducted GUS staining. Staining under normal conditions resulted in the appearance of blue stains in cotyledon, hypocotyl stele, leaves, root stele, and at the junction of the lateral root, sepal, filament, tissues between style and stigma, and tissue between silique and silique petiole (Figures 9A–J). Thus, the localization of *HsCBL8* was found similar to that of other *CBLs*. For instance, the presence of *AtCBL3* is in roots, leaves, siliques and seeds (Tang et al., 2012); *OsCBL2* is in the aleurone layer induced by GA (Hwang et al., 2005); whereas, *AtCBL1, AtCBL2, AtCBL3*, and *AtCBL9* were involved in seed germination and seedling and pollen development (Pandey et al., 2008; Li D. D. et al., 2013; Mähs et al., 2013; Zhou et al., 2015). Moreover, the overexpression of soybean *GmCBL1* promoted hypocotyl elongation under normal light conditions in *Arabidopsis* (Li Z.-Y. et al., 2013). *HsCBL8* may also involve light and GA response *cis* elements in its promoter sequence (Table 1), but few is known about this aspect and further study is needed.

Some genes from CBL family were considered stress responsive either directly or indirectly, by interacting with other genes (Kim, 2013). Therefore, the expression levels of the genes specifically responsive to salt stress, including *OsSOS*2/*OsCIPK24, OsNHX1*, and *OsSOS1* (Fukuda et al., 1999), were analyzed. Besides the genes expressed under salt-specific stress, microbe-associated molecular pattern-induced defense genes (*OsCIPK15*, Kurusu et al., 2010, ABA- and osmotic-stress-responsive genes (Rao et al., 2011), and drought-, high salt- and cold-stress-responsive (*OsDREB2A*, Dubouzet et al., 2003 genes were also analyzed. As evident in the results (Figure 4), the application of salt stress induced *HsCBL8* expression in the wild barley. Notably, the expression of HsCBL8 declined significantly at the first hour in root and increased significantly afterwards. Possibly, it takes time for plants adapting themselves to adverse environments, a process described as “shook” in some literatures (Munns and Tester, 2008; Shavrukov, 2013). The promoter analysis of *HsCBL8* revealed the presence of some stress response *cis* domains (Table 1), including ABA- and drought-response sequences, which was confirmed from the GUS activity analysis, as the promoter was activated by ABA, drought, and salt stress (Figure 9K), indicating the involvement of *HsCBL8* in upstream signaling pathway responding to these abiotic stresses.

ABA is widely considered as a stress-response phytohormone. A study on plants with CBL mutation revealed that *AtCBL9* was more sensitive to exogenous ABA and could regulate ABA bio-synthesis as the young seedlings of *cbl9* mutant accumulated additional ABA (Pandey et al., 2004). However, the plants overexpressing *AtCBL5* did not alter their response to ABA (Cheong et al., 2010), indicating that *AtCBL5* may regulate downstream genes independent from ABA. Moreover, we reported that AtCIPK15 could coordinate with several CBL proteins responding to ABA, including AtCBL1, 2, 3, 5, 8, and 9 (Batistic et al., 2012; Kim, 2013). These findings could suggest that *HsCBL8* might enhance tolerance against salt stress in rice by acting antagonistically to ABA biosynthesis or signaling. These observations were further confirmed when the overexpression of *HsCBL8* improved seed germination ratios in rice (Figure 6A). Furthermore, *HsCBL8* might regulate GA signaling against ABA.

In conclusion, *HsCBL8* derived from Qinghai-Tibetan wild barley was overexpressed in transgenic rice. This gene exhibited a significantly increased tolerance to high salinity through the systemic regulation of seed germination, proline accumulation, plasma membrane protection, and decreased overall Na^+^ uptake. No defect was observed in the germination, seedling, and reproductive stages of the transgenic plants under salt stress. This finding indicated that *H. spontanum* from Qinghai-Tibet plateau can provide a significant gene pool for cereal crop improvement (Ellis et al., 2000; Dai et al., 2012). Our study successfully demonstrated the involvement of *HsCBL8* in regulating various physiological processes that induce salt stress tolerance in rice. Our study could be used as a basis for further research on the potential functional attributes of *HsCBL8* in response to other abiotic stress factors and its interaction with other unknown factors.

## Author contributions

WG, LJ, and GZ conceived and designed the experiment and contributed reagents/materials/analysis tools. WG and TC conducted the experiments. WG, TC and NH analyzed the data. WG, TC, LJ and NH wrote the manuscript. WG, TC, NH, GZ, and LJ reviewed, edited and approved the manuscript.

## Funding

This work was supported by the Zhejiang Provincial Natural Science Foundation of China [LY13C130001]; the Science Foundation of Zhejiang Sci-Tech University [14042008-Y]; the Zhejiang Provincial Top Key Discipline of Biology [2012A03-C], and Jiangsu Collaborative Innovation Center for Modern Crop Production.

### Conflict of interest statement

The authors declare that the research was conducted in the absence of any commercial or financial relationships that could be construed as a potential conflict of interest.

## Supplementary material

Table S1 **Primers used in this program**.

Table S2 **Protein sequences of CBLs used in phylogenetic analysis**.

Figure S1 **Screening of homozygous T2 transgenic plants by PCR using: (A) 679 bp sequence of *HsCBL8*, and (B) RT-PCR analysis of the *HsCBL8* T3 transgenic lines using 575 bp sequence of *HsCBL8*, and *OsGAPDH* (BCBI ID: NM_001067432) as reference gene**. Six T3 seeds **(B)** come from T2 line T2-1-3 and T2-3-9 in **(A)**, respectively. M, DNA marker. CK^+^, *pCAMBIA1300-35S-HsCBL8*; CK^−^, ZH11.
