# Supplementary figures and images for: Characterization of Salinity Tolerance of Transgenic Rice Lines Harboring HsCBL8 of Wild Barley (Hordeum spontanum) Line from Qinghai-Tibet Plateau
Source: Front Plant Sci. 2016 Nov 10;7:1678. doi: 10.3389/fpls.2016.01678 (PMC5102885; doi:10.3389/fpls.2016.01678)

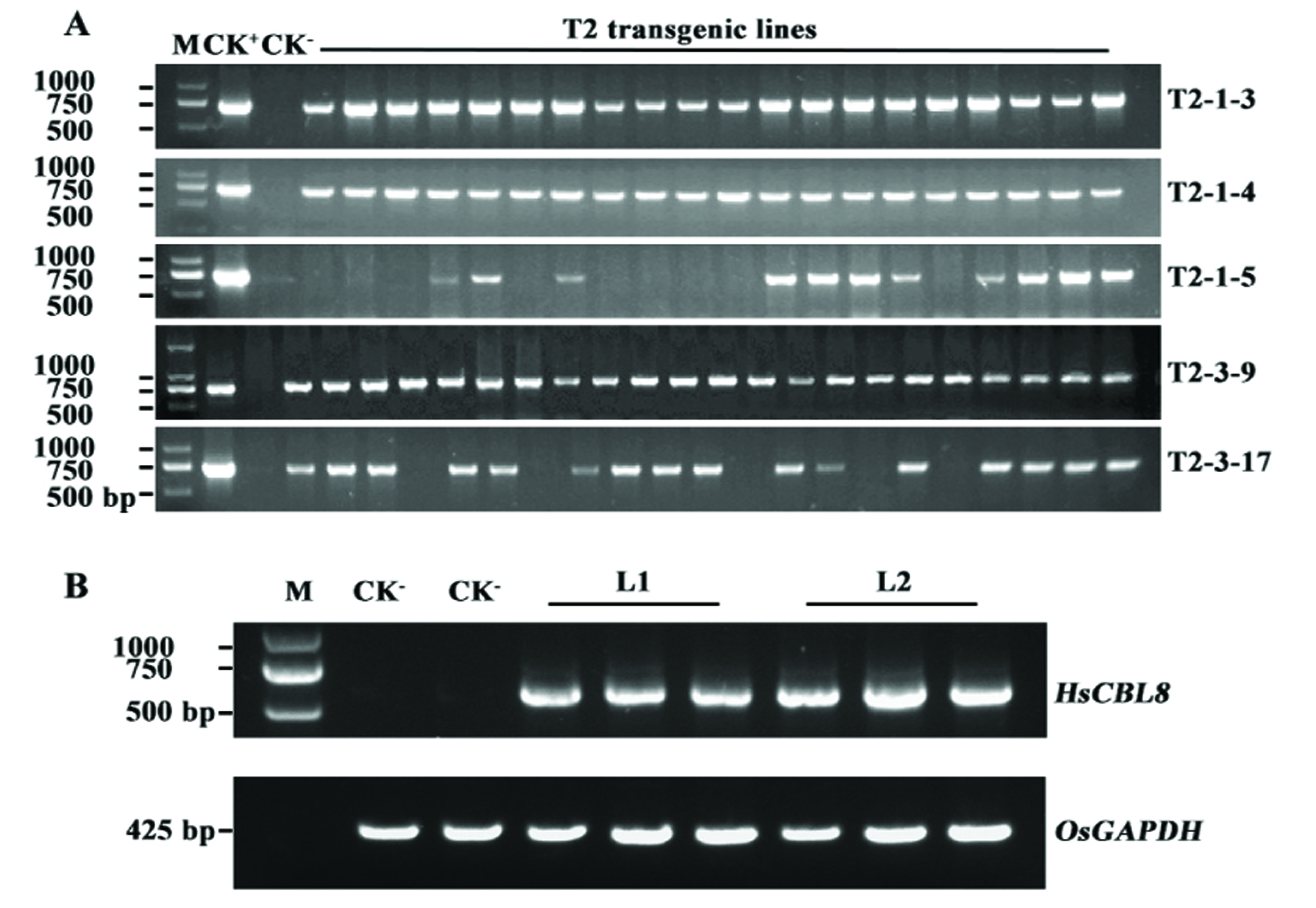

Supplement: Figure S1 — Screening of homozygous T2 transgenic plants by PCR using: (A) 679 bp sequence of HsCBL8, and (B) RT-PCR analysis of the HsCBL8 T3 transgenic lines using 575 bp sequence of HsCBL8, and OsGAPDH (BCBI ID: NM_001067432) as reference gene. Six T3 seeds (B) come from T2 line T2-1-3 and T2-3-9 in (A), respectively. M, DNA marker. CK+, pCAMBIA1300-35S-HsCBL8; CK−, ZH11. [file Image1.TIF]
